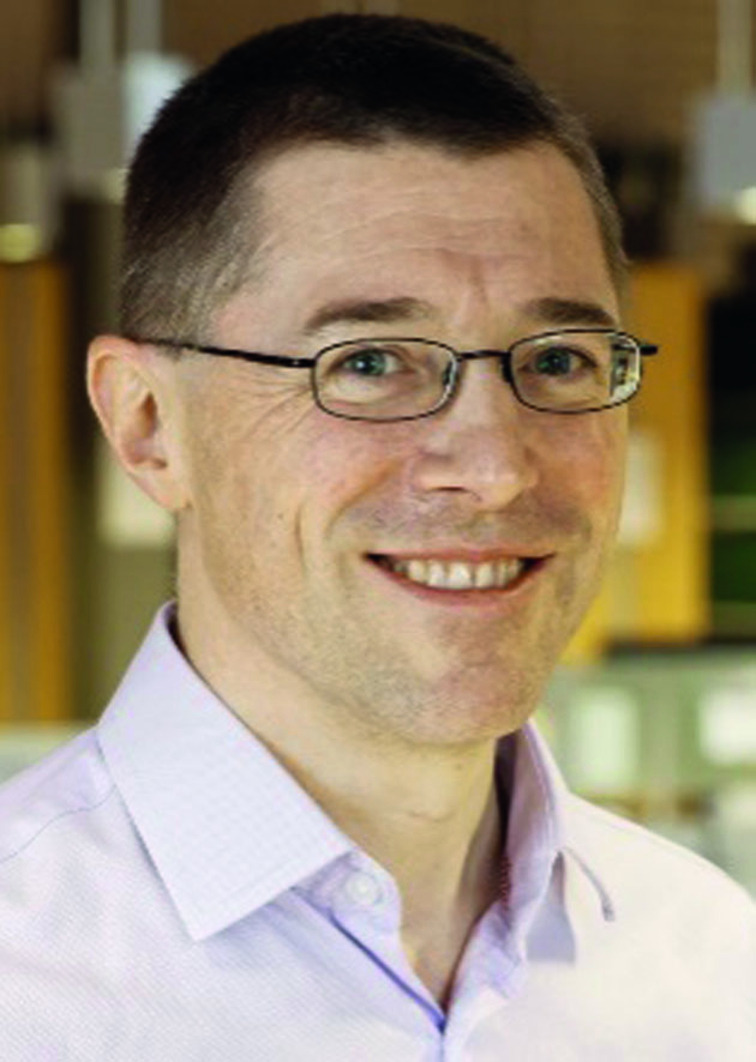# Methods for the Generation of Single‐Payload Antibody‐Drug Conjugates

**DOI:** 10.1002/cmdc.202500132

**Published:** 2025-03-19

**Authors:** Thomas Wharton, David R. Spring

**Affiliations:** ^1^ Yusuf Hamied Department of Chemistry University of Cambridge Lensfield Road Cambridge UK CB2 1EW

**Keywords:** Antibodies, Antibody-drug conjugates, Drug delivery, Drug-to-antibody ratio, Cytotoxicity

## Abstract

Antibody‐drug conjugates (ADCs) have emerged as a powerful form of targeted therapy that can deliver drugs with a high level of selectivity towards a specific cell type, reducing off‐target effects and increasing the therapeutic window compared to small molecule therapeutics. However, creating ADCs that are stable, homogeneous, and with controlled drug‐to‐antibody ratio (DAR) remains a significant challenge. Whilst a myriad of methods have been reported to generate ADCs with a DAR of 2, 4, and 8, strategies to generate DAR 1 constructs are seldom reported despite the advantages of low drug loading to tune ADC properties or to allow access to antibody‐antibody and antibody‐protein constructs. This concept article highlights the diversity of methods that have been employed to access single‐payload ADCs and explores the outlook for the field.

## Graphical abstract

Antibody‐drug conjugates (ADCs) have emerged as a powerful form of targeted therapeutic. However, despite the myriad methods to generate even‐integer drug‐to‐antibody ratio (DAR) ADCs, strategies to generate DAR 1 are seldom reported despite the property tuning advantages of low drug loading or in enabling access to antibody‐protein constructs. This Concept highlights the diverse methods employed to access single‐payload ADCs.

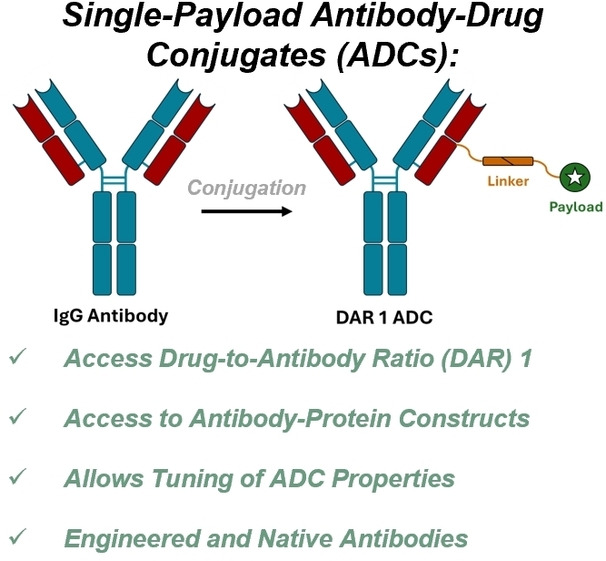

## 1 Introduction

**Scheme 1:**
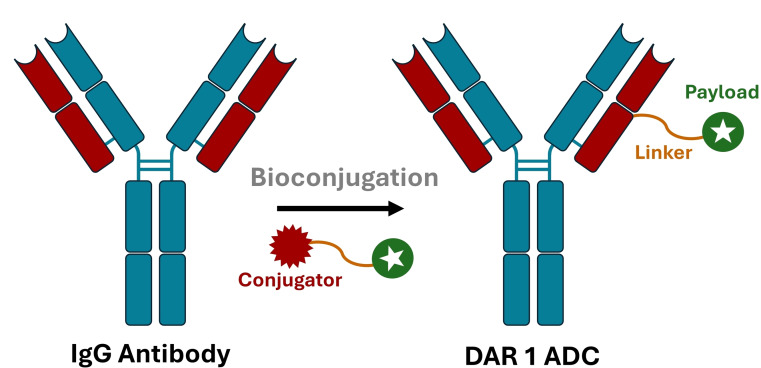
Conjugation of a single‐payload to an antibody enables access to DAR 1 ADCs and antibody‐protein constructs.

Antibody‐drug conjugates (ADCs) have emerged as powerful therapeutics over the last three decades, with interest in these biologics continuing to grow.[^1^, ^2^, ^3^, ^4^] ADCs are typically composed of three parts; a targeting antibody (normally IgG), a payload, and a linker (Scheme 1).^[2]^ The overall benefit of an ADC over its parent antibody alone is in combining the targeting ability and long half‐life of the antibody with the potency of small molecule cytotoxins to maximise their delivery to the desired cells whilst minimising off‐target toxicity,^[5]^ thus giving an improved therapeutic index compared to the drug or antibody components alone.^[6]^ ADCs are most commonly utilised for oncology[^2^, ^7^] and many ADC programs have been highly successful. The ADC field is experiencing seemingly exponential growth, with all but five of the 13 FDA‐approved ADC therapies authorised since 2019, and 350 more currently in clinical trials as of 2024.[^8^, ^9^]

The properties of an ADC vary greatly with the payload used, number of payloads, linker type, and method of linker conjugation to the antibody.[^2^, ^3^] In all currently FDA‐approved ADCs the payload is a highly potent cytotoxic drug. Approved ADC payloads include the tubulin binding maytansinoids (DM1 and DM4)^[10]^ and monomethyl auristatin E (MMAE), and the DNA disrupting calicheamicins^[11]^ and pyrrolobenzodiazepine (PBD) dimers,^[12]^ all of which show nano‐ to picomolar cell killing activity against a range of cancer cell lines.[^4^, ^9^] Non‐cytotoxic payloads, such as immune system stimulating agents^[13]^ and protein degraders[^14^, ^15^] have also been explored as well as radio labels,[^16^, ^17^] fluorescent probes,[^18^, ^19^] or combinations thereof that can be used as theranostics.^[20]^

The number of payload molecules appended to each antibody is crucial for its activity, pharmacokinetic profile, and propensity for aggregation.[^1^, ^21^] This value is most commonly reported as the drug‐to‐antibody ratio (DAR). Increasing DAR can lead to the ADC showing a higher clearance rate, uneven distribution, poor antigen binding, and higher toxicity,[^22^, ^23^, ^24^] though increasing the number of drugs that can be released can afford greater therapeutic benefit; therefore, a balance must be struck. Most ADCs utilise a DAR of 4–8 to maximise drug loading,^[22]^ however, drugs with picomolar activity, such as PBD dimers, cause off‐target toxicities at high loadings and so they are typically utilised in DAR 2 ADCs.[^4^, ^9^]

A myriad of methods have been developed for the bioconjugation of the linker‐payload component to antibodies. Of these, the most commonly utilised are direct attachment to native amino acid sidechains such as lysine and cysteine,[^25^, ^26^, ^27^, ^28^] genetically inserted unnatural amino acids,[^29^, ^30^] engineered glycans,[^31^, ^32^, ^33^] and affinity peptides.^[34]^

Conjugation methods typically result in even integer DAR values as most strategies cannot discriminate between the two symmetrical sides of an antibody. Therefore, there is increasing interest in accessing odd‐numbered DARs to expand the toolbox of methods and allow fine tuning of ADC properties. Despite the utility of DAR 1 ADCs to incorporate a single, highly toxic payload to reduce systemic toxicity, or allow the creation of bispecific protein‐antibody and antibody‐antibody conjugates,[^35^, ^36^] there are relatively few methods to access DAR 1 ADC species. This article highlights the diversity of strategies that have been developed to produce single‐payload ADCs and what work needs to be done to further the field. Whilst this article focuses on full‐antibody conjugates due to their clinical success, it is of note that single payload conjugation methods have also been described for antibody‐like constructs such as nanobodies and Fab fragments.[^37^, ^38^]

## 2 Key Advances

**Scheme 2:**
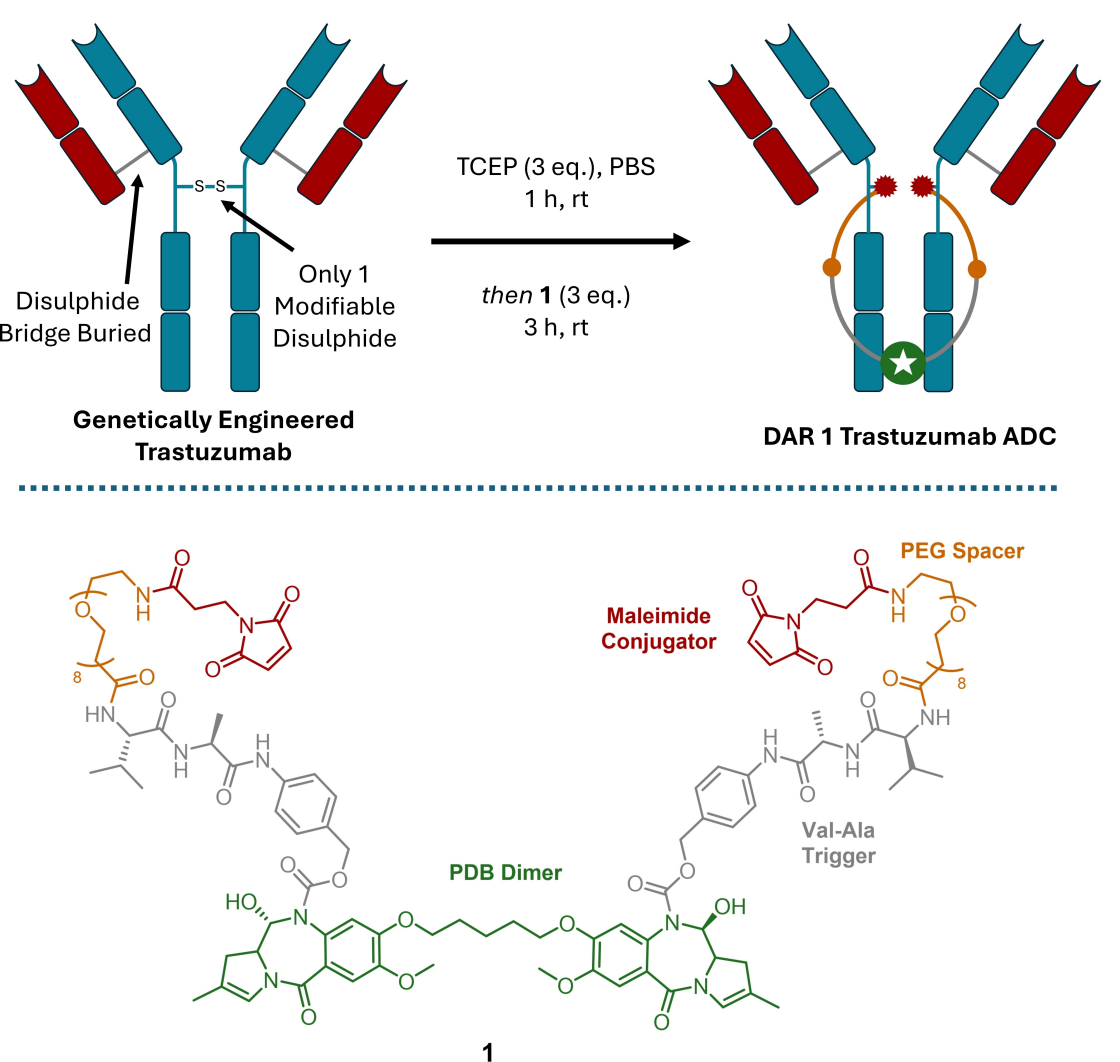
Genetic engineering ensures only two cysteines are accessible for bioconjugation. Using dual reactive linker **1**, a single payload can be added in a ‘loop’.^[39]^ TCEP=Tris(2‐carboxyethyl)phosphine; PBS=Phosphate buffered saline.

White *et al*. accessed a single payload PDB‐dimer ADC *via* genetic engineering of the HER‐2 targeting antibody trastuzumab (Scheme 2).^[39]^ A trastuzumab mutant was expressed with both pairs of light‐heavy chain disulphide bridges buried in the structure and one of the hinge region interchain disulphides removed. This left a single accessible interchain disulphide bond that was re‐bridged with three equivalents of a double maleimide functionalised PDB‐dimer linker (**1**), giving 90 % conversion to the DAR 1 species. This use of a doubly reactive linker is akin to the work of Lee *et al*. who described a double dibromopyridazinedione linker to re‐bridge two pairs of native interchain disulphide bonds with a single linker, thus affording DAR 2 conjugates when all four disulphide bonds were re‐bridged.^[40]^

As linker **1** was symmetrical, a valine‐alanine (Val‐Ala) dipeptide was incorporated on either side of the PDB‐dimer to allow selective free drug release upon peptide bond cleavage, caused by cathepsin enzymes. The DAR 1 species showed a minimum effective dose of 0.6 mg/kg, unsurprisingly twice that of a DAR 2 analogue (0.3 mg/kg), whilst exhibiting lower hydrophobicity, higher tolerability in mice, and slower clearance suggesting that the lower drug loading was beneficial.

**Scheme 3:**
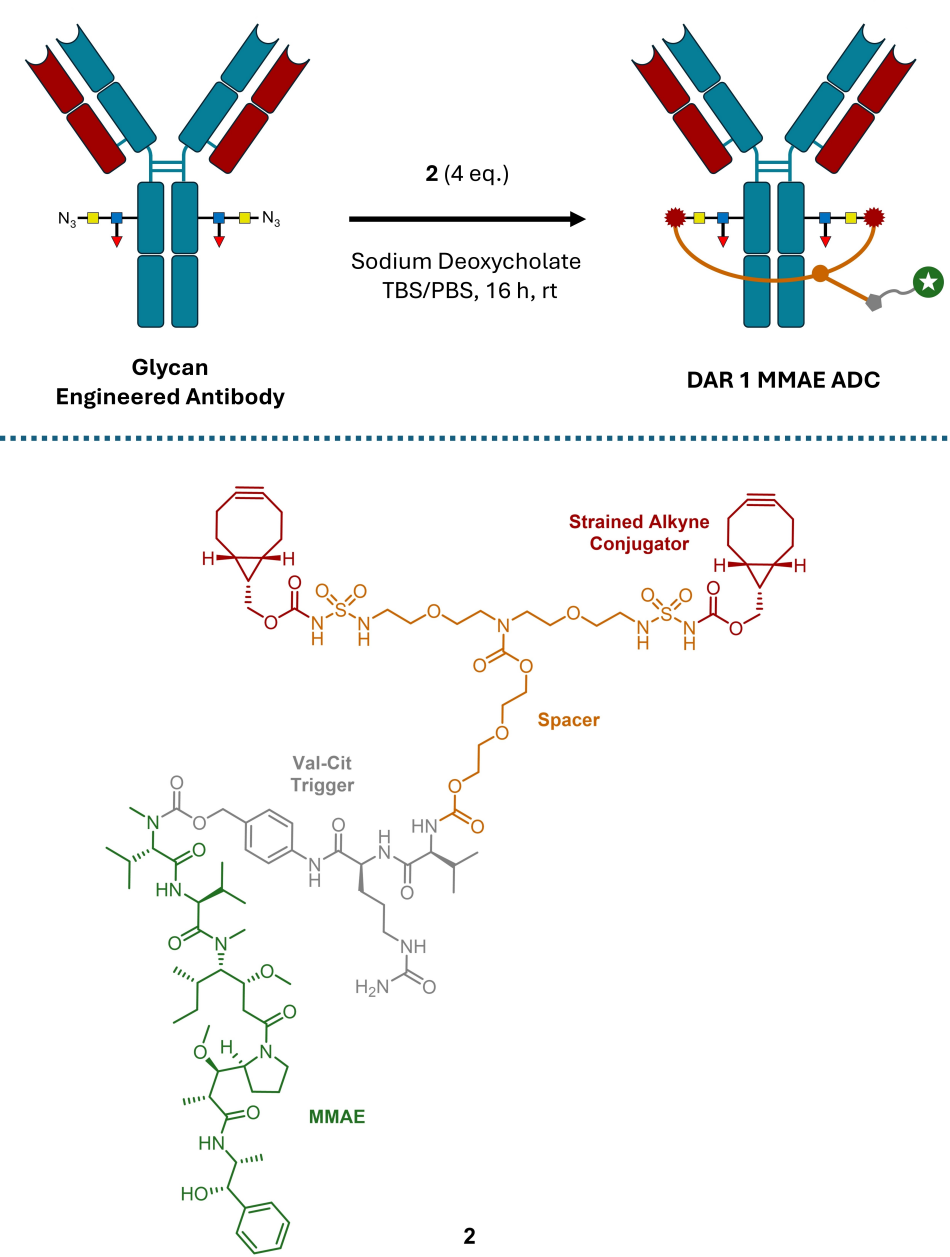
Glycan engineering installed reactive azide handles which can undergo SPAAC with a bis‐reactive linker to form a ‘loop’ with a single payload attached.^[31]^ TBS=Tris buffered saline; PBS=Phosphate buffered saline.

Utilising GlycoConnect^TM^ technology developed in‐house, De Bever *et al*. disclosed a glycan engineering approach to selectively modify two sites on trastuzumab to install azide handles (Scheme 3).^[31]^ By linking the two azides with a bis‐reactive alkyne linker *via* strain promoted azide‐alkyne click (SPAAC) reactions, a loop was formed with a single payload attached (inspired by the approach of White *et al*.^[39]^). The glycan engineering relies on the use of an endoglycosidase to trim the natural sugars before the azide handle is installed by a glycosyltransferase. As the sugars are engineered selectively, the method avoids the need for genetic engineering of the antibody.

The method was developed on linkers with an MMAE payload and valine‐citrulline (Val‐Cit) dipeptide release trigger where the length and hydrophilicity of the spacer between the two alkyne click handles and the payload was optimised for click efficiency. Four equivalents of PEG‐based acylsulfamide linker **2** was found to achieve 91 % desired azide crosslinking whilst minimising the amount of over‐addition (where each azide reacts with two different alkyne linkers instead of both alkynes on a single linker; 5.6 %) and unreacted starting azide‐antibody (2.5 %). The authors further improved the ratio of species by removing the over‐addition product via capturing the unreacted alkyne handles on tetrazine beads, increasing the proportion of desired azide crosslinked single‐payload ADC up to 98 %.

The method was then shown to be compatible with other potent cytotoxic payloads such as a PDB dimer and a calicheamicin variant, in each case affording ADCs of DAR 0.9–1.0. As expected the DAR 1 ADCs showed IC_50_ values twice that of DAR 2 analogues when tested in vitro.

**Scheme 4:**
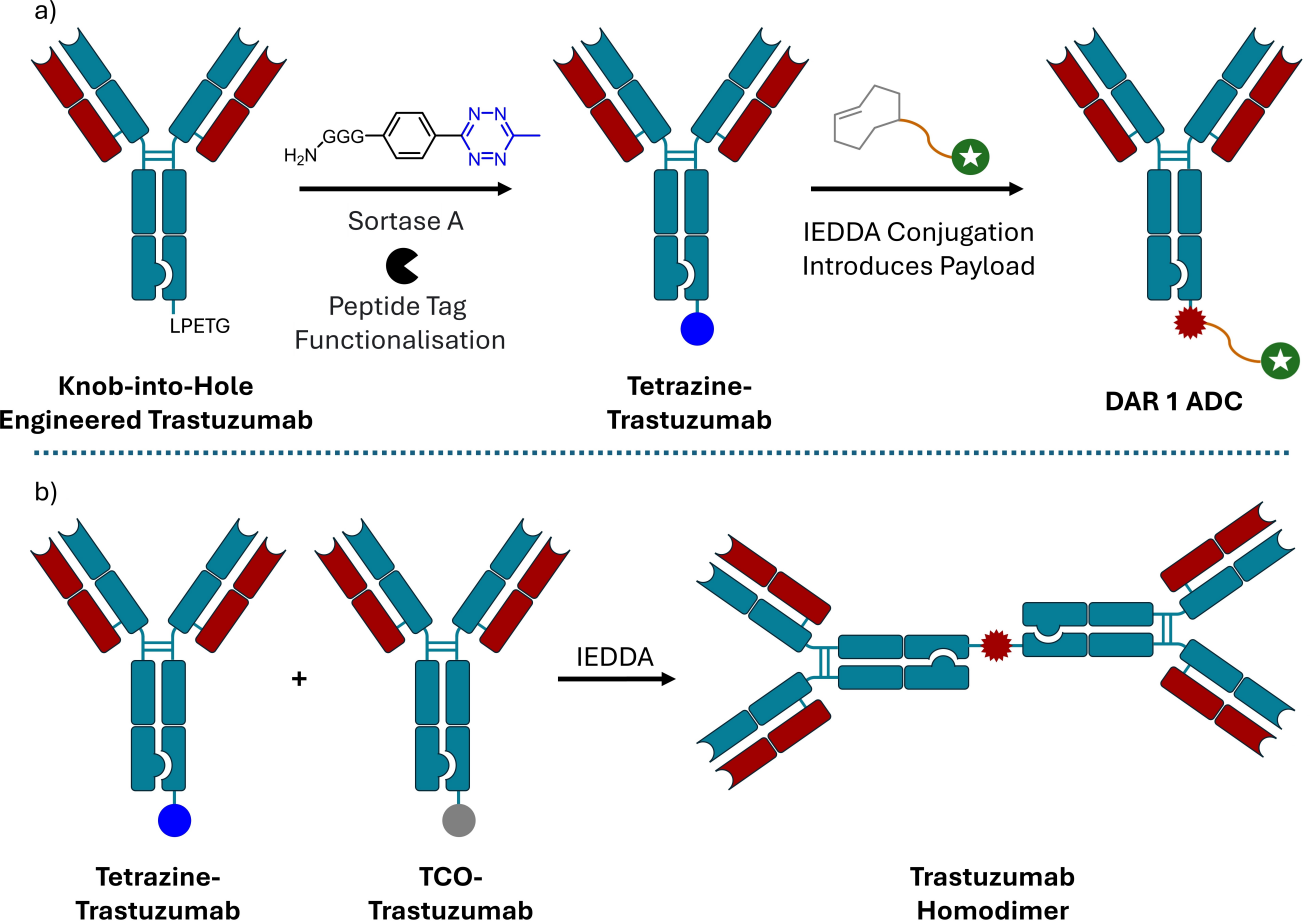
‘Knob‐into‐Hole’ antibody engineering allows for single site‐specific modification of a heavy chain C‐terminus.^[41]^ a) Example work flow. Sortase A is used to functionalise a peptide tag with a tetrazine handle that can then undergo IEDDA with a TCO‐payload. b) Combining tetrazine‐ and TCO‐functionalised antibodies allows the creation of antibody‐antibody conjugates via an IEDDA reaction.

Bruins *et al*. used a ‘Knob‐into‐Hole’ (KiH) approach to differentiate the two halves of an antibody, thus allowing for a single reactive handle to be incorporated into only one of the heavy chains for single‐payload attachment (Scheme 4).^[41]^ The KiH approach relies on genetically modifying the heavy chains, in which one batch contains a section of amino acids with increased side‐chain length (creating a ‘knob’) and a second batch contain a corresponding sequence with decreased side‐chain length (creating a ‘hole’). Thus, when mixed the heavy chains will arrange in pairs knob‐into‐hole to maximise binding. Bruins *et al*. engineered a trastuzumab‐based KiH antibody, incorporating short peptide tags on the *C*‐terminus of the ‘hole’ batch of heavy chains that could be recognised by enzymes, such as sortases. These tags then allowed for tetrazine or trans‐cyclooctene (TCO) handles to be appended onto the heavy chain that could be used in inverse‐electron demand Diels−Alder (IEDDA) reactions to conjugate payloads. IEDDA reactions were used to conjugate a fluorophore (TAMRA), protein (Interleukin‐2), and antibody fragment (variable region of UCHT1) onto the KiH with HPLC analysis suggesting good conversion to single payload species in all cases. Protein A affinity column chromatography was used after each step to purify out unreacted small molecules and enzymes. Finally, a homodimer antibody‐antibody conjugate was produced using a complementary pair of tetrazine‐ and TCO‐containing KiH antibodies, a result difficult to achieve through other means, though with low conversion. Biological studies to assess if activity and targeting were retained were not disclosed.

**Scheme 5:**
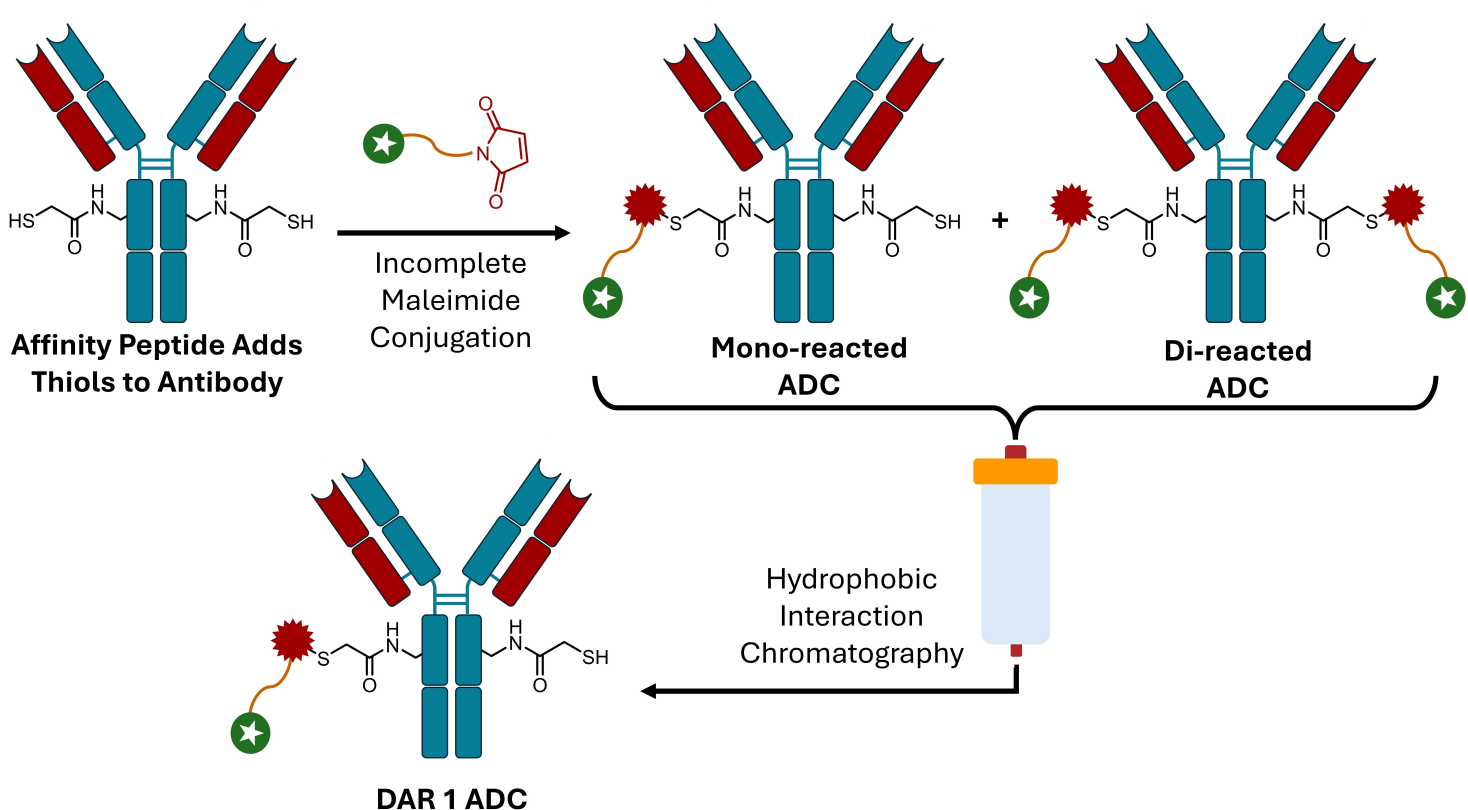
Incomplete conjugation leads to a mixture of mono‐ and di‐reacted ADC. DAR 1 species can be obtained by purifying out the mono‐reacted species.^[42]^

Matsuda *et al*.^[42]^ and Dovgan *et al*.^[43]^ utilised selective purification to attain DAR 1 ADCs. The method employed by Matsuda *et al*.^[42]^ relied on simply using low equivalents of linker to restrict a DAR 2 conjugation from reaching completion. Using an affinity peptide approach previously reported by the group termed AJICAP^TM^,^[44]^ free thiols were installed specifically on each lysine 248 in the heavy chains of trastuzumab (Scheme 5). These thiols were then reacted with an unreported amount of maleimide‐Val‐Cit‐MMAE linker to give a mixture of mono‐ and di‐reacted ADC. The species were then separated out using hydrophobic interaction chromatography (HIC) to give a pure DAR 1 product. As the di‐reacted species is the major product of the conjugation, the DAR 1 ADC recovery is relatively low, however, as the only requirement to carry out this method is a HIC column, it is applicable to any ADC conjugation strategy.

**Scheme 6:**
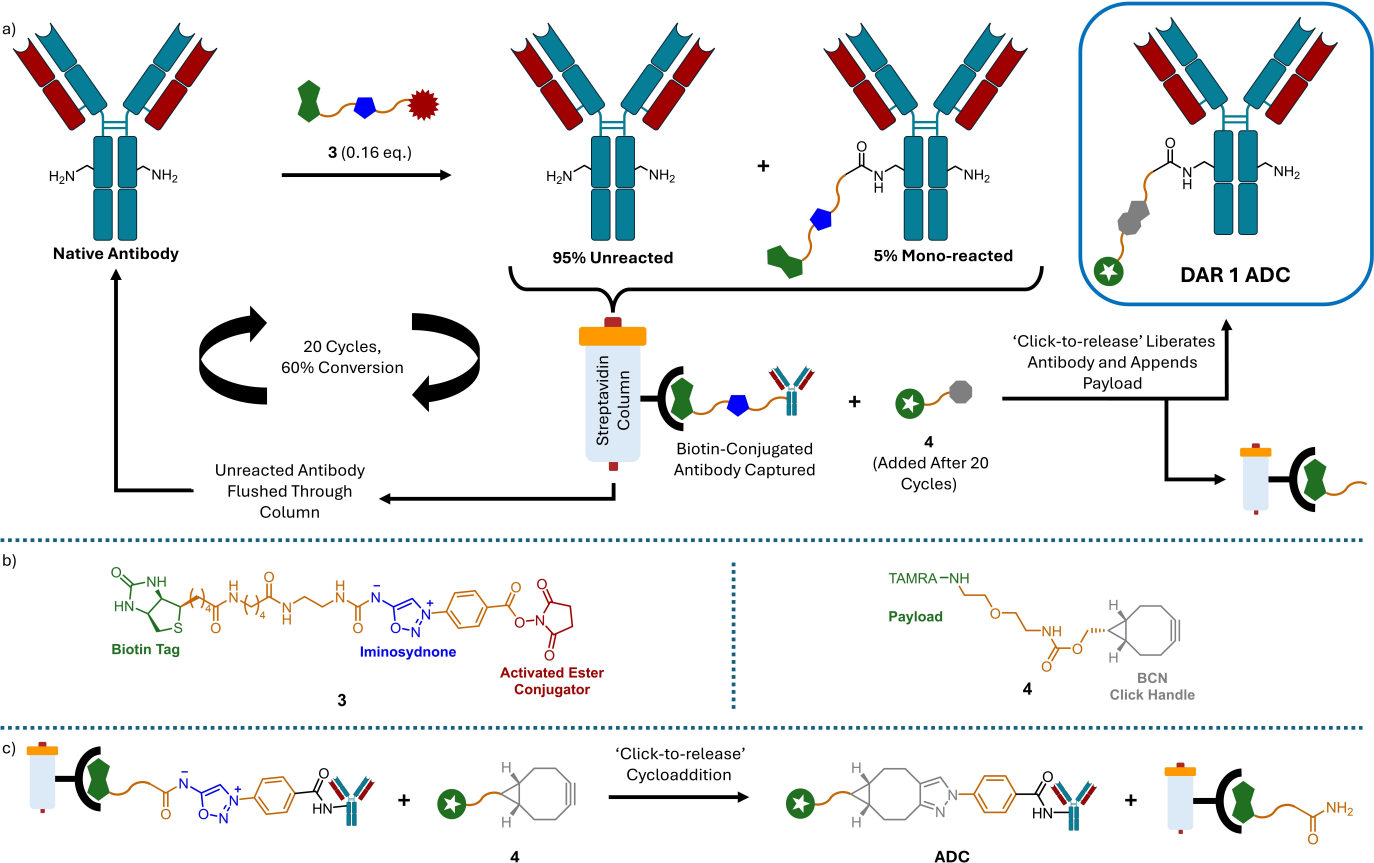
Repeated low conversion bioconjugation and starting material recycling allows for the controlled accumulation of single‐payload ADCs.^[43]^ a) Reagent addition cycles followed by purification with a streptavidin affinity column allows unreacted antibody to be recycled whilst capturing mono‐reacted ADC. Treatment of the column with strained alkyne **4** swaps the biotin tag for a payload and releases the desired ADC. b) Structures of the biotin tag and BCN‐Payload click handle. c) Full scheme of the ‘click‐to‐release’ cycloaddition. BCN=Bicyclononye.

Dovgan *et al*.^[43]^ also used low conversion bioconjugation to produce DAR 1 ADCs but opted for repeated reagent addition cycles followed by purification to slowly increase the presence of single‐payload species (Scheme 6). The researchers utilised activated‐ester linker **3** to stochastically label lysine residues. By only using 0.16 equivalents of **3** the researchers were able tominimise double addition, ensuring that only singly modified antibody and unmodified antibody were produced. As linker **3** contained a biotin tag, the modified antibody could be trapped on a streptavidin affinity column with any unmodified antibody washed out and collected for subsequent rounds of reagent addition and purification. As each round produced 5 % modified antibody the sequence was carried out 20 times to afford a 60 % conversion. As the linkage between the antibody and the biotin tag contained an iminosydnone ‘click‐to‐release’ handle^[45]^ the modified antibody could be released from the affinity column by adding strained bicyclononye (BCN) **4**. Furthermore, as the strained alkyne can include further functionality the addition of the alkyne simultaneously detaches the antibody from the column and appends a payload to it. To the end, the production of DAR1 MMAE and TAMRA conjugates was showcased. The whole process could be efficiently carried out using a semi‐autonomous flow set up using syringe barrels and 3D‐printed parts, for which schematics and building instructions were well described. Whilst this technique was developed primarily to produce mono‐disperse conjugates via the myriad lysine attachment sites, it could be used to produce homogeneous conjugates if a site‐specific conjugation method was utilised.

**Scheme 7:**
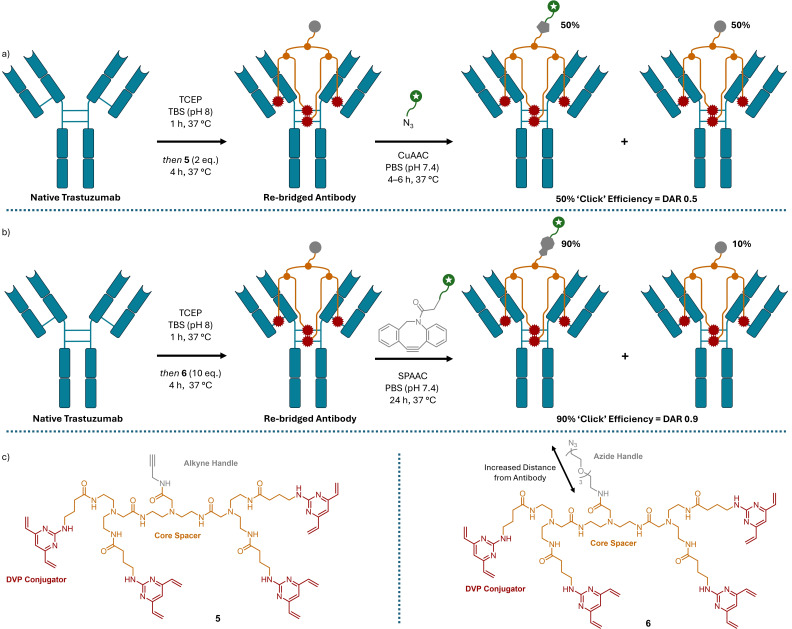
The four reactive DVP motifs of TetraDVP enable the re‐bridging of all four disulphide bonds within a single antibody. a) Initial work using CuAAC led to poor ‘click’ efficiency affording DAR 0.5 ADCs.^[47]^ b) Using SPAAC enabled an increase in DAR to 0.9.^[48]^ c) Structures of the TetraDVPs used. Linker core length was also found to impact the conjugation and click rates. TCEP=Tris(2‐carboxyethyl)phosphine; TBS=Tris buffered saline; PBS=Phosphate buffered saline.

With the aim to access DAR 1 ADCs with high bioconjugation conversion without the need for antibody or glycan engineering, or purification, Spring and coworkers extended their previously described divinyl pyrimidine (DVP)[^27^, ^46^] cysteine re‐bridging bioconjugation strategy to produce so‐called TetraDVP; a construct that can re‐bridge all four disulphide bonds within a single IgG1 antibody at once.[^47^, ^48^] This approach greatly lowers the possibility of re‐bridging ‘half‐antibody’ species that was seen in previous DVP studies^[27]^ and as the linker contains a single modifiable handle it allows access to DAR 1 ADCs. In their initial work, Dannheim *et al*. developed the TetraDVP concept achieving good bioconjugation conversion on trastuzumab and brentuximab with two equivalents of linker, making the process atom economic (Scheme 7a).^[47]^ The alkyne handle of TetraDVP **5** enabled post functionalisation of the TetraDVP conjugate and to this end AlexaFluor 488 and MMAE were appended *via* copper‐catalysed azide‐alkyne click (CuAAC) reactions. TetraDVP conjugates of trastuzumab were found to retain binding affinity to HER2, showed comparable stability to a DAR 4 mono‐DVP analogue, and a trastuzumab‐AlexaFluor 488 conjugate was successfully utilised as a diagnostic probe in HER2‐positive cell lines. Whilst the CuAAC reaction with AlexaFluor 488 proved high yielding, the size of the azide‐MMAE linker used hindered the conversion to around 50 %, leading to a DAR of 0.5, despite attempts to optimise the reaction using up to 100 equivalents of azide‐MMAE. Further TetraDVP linkers were synthesised incorporating two, three, or four alkyne handles with the intention to produce ADCs with DARs 2, 3, and 4. However, as before the CuAAC reactions failed to reach completion, with the resulting ADC DARs recorded as 1, 1.6, and 2.4, respectively.

King *et al*. aimed to improve the DAR by swapping the alkyne on the TetraDVP core to an azide (**6**) to allow the payload to be appended *via* SPAAC instead of CuAAC reactions (Scheme 7b).^[48]^ This subtle change vastly improved the ‘click’ efficiency and allowed the DAR to be increased to up to 0.9 without the need for chromatographic purification. The study also highlighted that changing the distance between the DVP head groups impacted the bioconjugation with longer linkers improving the extent of conjugation.

## 3 Summary and Outlook

This concept article highlights the diverse strategies that can be employed in the synthesis of single‐payload antibody drug conjugates, ranging from genetic and glycan engineering to selective purification and multibranched linker systems.

White *et al*. and De Bever *et al*. used genetic and glycan engineering (respectively) to allow the introduction of a single pair of chemically reactive handles to the heavy chains of the antibody. Via the use of doubly reactive linker‐payload scaffolds, the groups where able to generate single‐payload species by creating a ‘loop’ between these heavy chain fragments. Bruins *et al*. used genetic engineering to create complementary ‘Knob‐into‐hole’ heavy chain pairs. One of the pair was also modified to contain a protein tag that could be recognised by enzymes to allow a payload to be appended. These methods can lead to high conversion to single‐payload species once the antibody of choice has been suitably engineered.

Matsuda *et al*. and Dovgan *et al*. opted to separate differing DAR species via column chromatography to yield pure single‐payload species. This strategy has the advantage of being applicable with any established bioconjugation method but can lead to low ADC recovery.

Finally, Dannheim *et al*. and King *et al*. developed tetra reactive linkers to take advantage of the selectivity possible utilising the natural disulphide bonds present in IgG antibodies. Through the use of branched linkers, single payload ADCs could be accessed with native off‐the‐shelf antibodies, though the extent of conversion was affected by the nature of the payload utilised.

Controlling precisely the number of payloads in a homogeneous and site‐specific way allows for greater control of ADC properties. For probe systems or extremely toxic molecules there is a need to produce ADCs with a single payload. The methods reported for single‐payload conjugation are limited but recent work has greatly advanced the area, which will bring major benefits to ADC programs by expanding the methods available.

The future of the field lies in utilising the methods to produce a variety of DAR 1 ADCs and antibody‐protein/antibody‐antibody constructs that are not accessible by other means. For the generation of effective ADCs the combination of payload, antibody, and linker are crucial. Highly toxic payloads can benefit from incorporation into DAR 1 ADCs to improve the pharmacokinetics and pharmacodynamics of therapeutics. Furthermore, by producing diverse payload‐antibody combinations improved therapeutics can be accessed.

As Bruins *et al*. showed, conjugating an antibody with a single reactive handle allows for the creation of antibody‐antibody constructs via a single linker. Further use of antibodies conjugated with a single reactive handle will allow diverse antibody‐antibody combinations to be synthesised, opening up new highly targeted bispecific therapies leveraging the combined specificity of each antibody. In a similar manor, single payload attachment will allow for precise 1 : 1 antibody‐protein constructs to be made which could be used to probe biological processes by utilising orthogonal binding of an antibody and protein. Alternatively, DAR 1 antibody‐protein constructs could be beneficial if size and activity warranted use of a single protein. Furthermore, a controlled delivery of a protein to a specific cell could also be afforded with a cleavable linker, akin to classical ADCs.

Whilst all the methods described above allowed access to single‐payload species, many require complex antibody or process manipulation which limits DAR 1 ADC recovery and scale‐up. Therefore, highly yielding conjugations which do not require chromatographic purifications or antibody manipulation are desired and should be further pursued.

To enable ADC properties to be effectively tuned, research into accessing other classically challenging DARs (such as 3) should be carried out via considered linker design and site selective conjugation. With robust methods developed, ADCs with precise DARs of 1–8 could be easily accessed, allowing for the drug loading to be tuned to balance the ADC's properties.

Overall, a variety of strategies for the generation of single‐payload ADCs have been described, allowing access to proof‐of‐concept DAR 1 ADCs and antibody‐protein/antibody‐antibody constructs for use as potent therapeutics and diagnostics tools. Further development of the methods and their translation to generate clinically relevant constructs will showcase the importance of these strategies in producing next‐generation antibody‐based therapeutics.

## Conflict of Interests

The authors declare no conflict of interest.

## Biographical Information

*Thomas Wharton undertook his undergraduate study at the University of Leeds, graduating with a MChem which included a year in industry on the RiCH Internship program at Roche, Basel. Thomas carried out his PhD studies with Prof. David Spring at the University of Cambridge working on small molecule release systems and antibody‐drug conjugates. He is currently continuing this research as a postdoctoral research associate in the Spring group*.

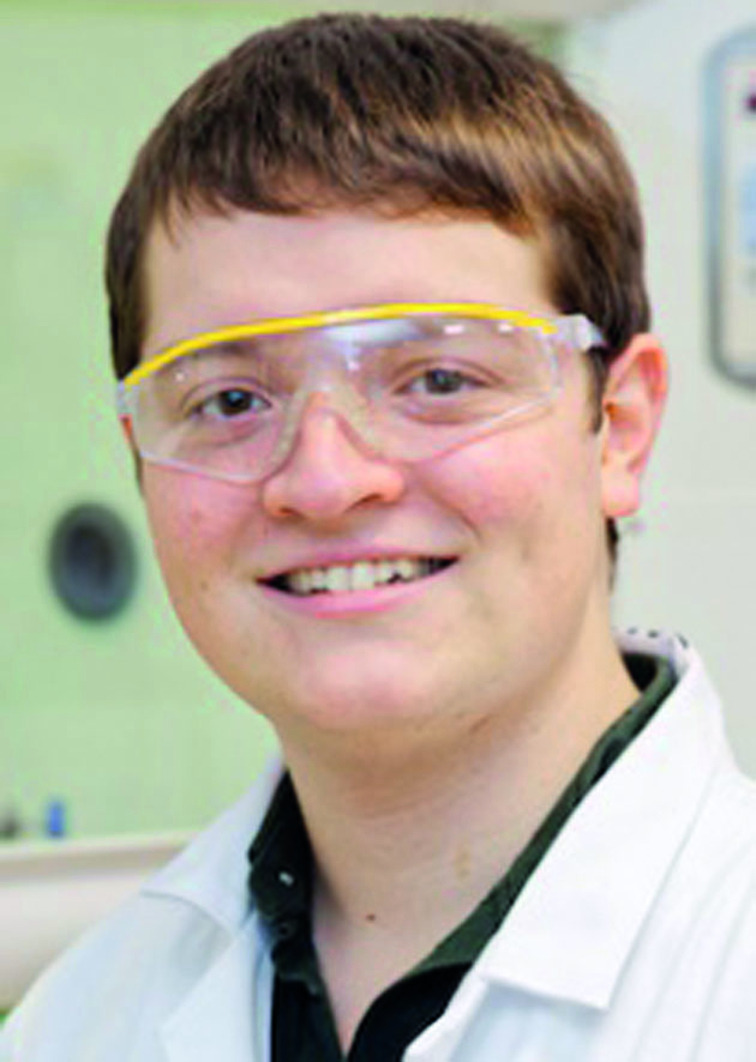

## Biographical Information

*David Spring is currently Professor of Chemistry and Chemical Biology at the University of Cambridge within the Chemistry Department. He received his DPhil (1998) at Oxford University under Sir Jack Baldwin. He then worked as a Wellcome Trust Postdoctoral Fellow at Harvard University with Stuart Schreiber (1999‐2001), after which he joined the faculty at the University of Cambridge. His research programme is focused on the use of chemistry to explore biology*.